# Dural Tear During Thoracic Laminectomy Using Craniotome With Footplate Attachment

**DOI:** 10.7759/cureus.14893

**Published:** 2021-05-07

**Authors:** John K Houten, David H Harter, Amit Y Schwartz

**Affiliations:** 1 Neurosurgery, Hofstra Northwell School of Medicine, New York, USA; 2 Division of Pediatric Neurosurgery, New York University School of Medicine, New York, USA; 3 Division of Neurosurgery, Maimonides Medical Center, Brooklyn, USA

**Keywords:** complication, craniotome, drill, dural tear, footplate, incidental durotomy, laminectomy, laminoplasty, spinal surgery

## Abstract

Laminectomy can be accomplished using the craniotome with a footplate attachment, and the technique has been advanced as a superior alternative to using a high-speed drill-driven burr and Kerrison rongeurs. Laminectomy can be accomplished more rapidly and with less bone destruction, an advantage when planning laminoplasty. There is, however, scant literature describing complications of dural laceration using this technique. A 48-year-old male underwent T7-10 laminectomy for resection of an intramedullary spinal cord tumor. During the upward cut of the hemi-lamina at T7-9, a dural laceration occurred that proved not amenable to direct suture closure. The dural was closed with a dural patch placed along the inner surface of the dura and a fat graft on the outer surface with adjunctive use of a lumbar drain. While the footplate laminectomy technique has merits touted in prior publications, including the ability to open the spinal canal quickly at numerous levels and an enhanced ability to achieve an osteoplastic laminoplasty, surgeons should be cognizant of the risk of associated dural laceration. We believe that it is important to emphasize that the initial placement of the lip of the footplate must be well-seated under the inferior aspect of the lowest lamina and over the ligamentum flavum and that the footplate should not be directed beyond the border of the laminae and facet, as this can result in dura and root injury.

## Introduction

Laminectomy is frequently performed to decompress neural compression resulting from degenerative disease and to expose the dura for tumor resection. Early methods to access the spinal canal involved the Doyen and Gigli bone saw, hand-powered drills, and osteotomes [1-3]. However, introduction of surgical tools into the spinal canal, particularly in the cervical region in the presence of severe stenosis, can result in neurological injury. The introduction of powered drills to thin the lamina was an important development in addressing this concern [4]. Hirabayashi et al. described the use of a customized thin-plate Kerrison rongeur and air-powered drill in the execution of open-door laminoplasty [5]. Most contemporaneous procedures to posteriorly decompress cervical canal employ the use of high-speed drills that thin or just traverse the lamina, allowing thin-footed 1 or 2 mm Kerrison rongeurs to complete the decompression [6,7].

Postoperative kyphotic deformity is a concern after cervical laminectomy. To deter the development of postlaminectomy kyphosis following cervical laminectomy, fusion or laminoplasty is advocated by many authors in adults. In children following resection of intramedullary spinal cord tumors (IMSCTs), osteoplastic laminoplasty is reported to deter development of postlaminectomy kyphosis [4,8-12]. The use of the high-speed drill for laminectomy may interfere with laminar replacement as the relatively large bony gap that is created can hamper various techniques that replace and fixate the posterior elements.

Some authors have advocated for the use of the craniotome with a footplate attachment to perform laminectomy [11,13-15]. Favorable aspects of the technique include that the small profile of the footplate is comparable to the 2 mm Kerrison rongeur, a bone-cutting mechanism that minimizes heat generation that can potentially be transmitted to the neural elements that might cause thermal injury, and its ability to offer faster laminectomy that minimizes in-and-out hand movements around the spinal canal [16]. The technique does not require threading a wire under the lamina prior to bone cutting as with the Gigli and threadwire saws [4]. We present the first case of laceration created at the level of a thoracic root using craniotome with footplate technique during laminectomy to treat an IMSCT.

## Technical report

**Figure 1:**
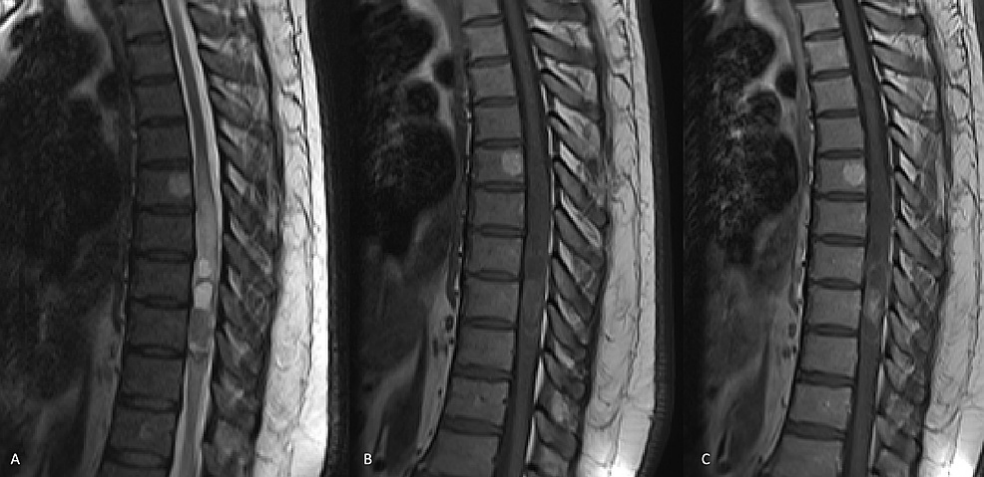
A thoracic spine MRI in a 48-year-old male with progressive myelopathy. Images in the sagittal plane in T2 (A), T1 without contrast (B), and T1 with contrast (C) demonstrating an enhancing intramedullary lesion in the T7-9 area with perilesional cord edema, a cyst, and a suggestion of a hemosiderin cap, the overall appearance of which was considered most likely to represent ependymoma. A hemangioma is incidentally noted in the T6 vertebral body. MRI: magnetic resonance imaging

A 48-year-old male presented with a five-month history of a sensation of numbness in the lower abdomen, tingling and numbness in the right leg, weakness in the left leg, and a sense of gait unsteadiness. He reported no problem with bowel, bladder, or sexual function. After a trial of physical therapy that did not produce symptom relief, he underwent magnetic resonance imaging (MRI) of the lumbar spine that showed an intraspinal tumor that upon additional thoracic spine MRI with and without contrast demonstrated a large and enhancing IMSCT centered at the T8/9 disc space level with perilesional edema and some low-signal-intensity hemosiderin rim considered most likely to represent an ependymoma (Figure 1).

**Figure 2:**
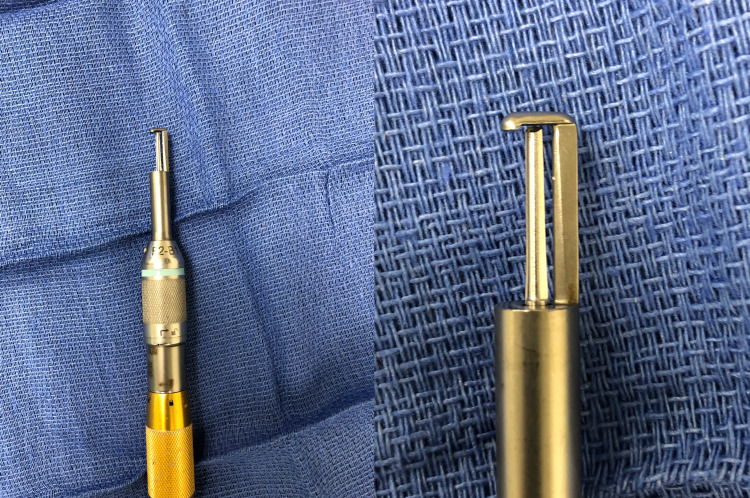
A Midas Rex drill with the craniotome and footplate attachment. The Midas Rex drill footplate attachment, seen in close-up on the right, acts as a guard between the cutting bit and dura.

Upon neurological examination, he had no abnormality in the mental status or cranial nerves, but there was 4-/5 weakness affecting all the muscle groups of the left leg and diminished proprioception in the right leg. Reflexes were normal in the arms but 4+ in the legs with bilateral ankle clonus and bilateral upgoing toes. He was indicated for surgical management. Following exposure of the T6-11 lamina and fluoroscopic-level verification, the T10/11 and T6/7 ligamentum flavum was partially resected from the interlaminar space using a fine Kerrison rongeurs to exposure the dura. The Midas Rex drill (Medtronic Inc., Minneapolis, MN) with the cranial footplate attachment was then placed underneath the lamina edge at T10 and moving cephalad the lamina were cut on either side (Figure 2).

**Figure 3:**
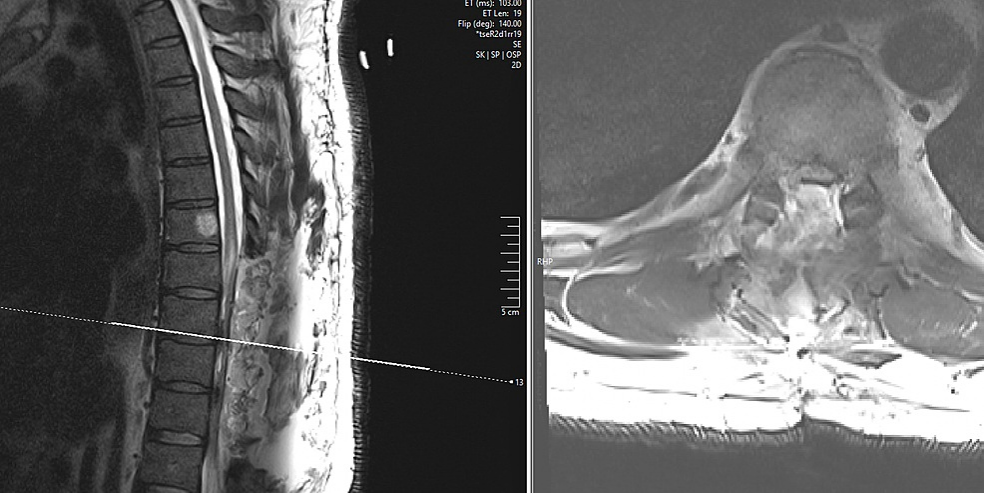
MRI in T2-weighted sequences one week following gross total resection of a T7-9 intramedullary spinal cord ependymoma. Images in sagittal (left) and axial (right) planes demonstrate resection of the intramedullary mass and mixed intensity fluid impressing upon the dura posteriorly, thought to be a mixture of CSF and blood product. Fluid appears to extend into the right lateral T8/9 recess where the dural tear had occurred, though no definitive defect in the dura is visible on MRI. MRI: magnetic resonance imaging; CSF: cerebrospinal fluid

Upon elevation of the T7-10 lamina, a longitudinal laceration of the dura on the left was appreciated appearing to have started upon catching the dura of the T9 nerve root sleeve. A midline durotomy was created, and under the microscope the tumor was exposed using a midline myelotomy. A gross total resection was achieved. Neuromonitoring demonstrated declination of the left motor potentials during resection without change in the somatosensory potentials, but there were no changes associated with the root avulsion during exposure. A dural graft was applied internally to the left dural laceration and was reinforced externally with a fat graft to fill the lateral extradural space. A lumbar drain was placed and the patient was initially kept at flat bed rest. Postoperatively, there was increased weakness of the left leg strength that was improving at the two-month follow-up but not fully to baseline. Final pathology was World Health Organization grade II ependymoma. Follow-up MRI one week following surgery demonstrated no enhancement suggestive of residual tumor and dorsal compression of the dura from fluid (Figure 3).

## Discussion

Laminectomy performed using the high-speed drill to progressively thin the lamina is a widely accepted technique used by spinal surgeons worldwide, with the intent of minimizing the risk of iatrogenic neurological injury by minimizing the encroachment within the spinal canal [7]. The technique, while generally executed safely, still carries the risk of dural tear and neurological injury [17]. Some authors have advocated the use of ultrasonic bone scalpel in laminectomy, a device that cuts bone with ultrasonic vibration under continuous cooling with water, arguing that the device does not cut soft tissues such as dura and is, thus, theoretically safer than the use of a high-speed burr [18]. Acceptance of the technique has been limited, however, in part out of concern for thermal injury that may result from inadequacy of the cooling mechanism, high cost of the equipment, the need for a learning curve, difficulty acclimating to a relatively bulky handpiece, and few reports showing any significant reduction of complications including dural tear or neurological injury [19,20].

Neurosurgeons are trained and experienced in using a craniotome with a footplate that enables large craniotomies to be created rapidly and safely, and some surgeons have applied this technique to the performance of laminectomy. Elia et al. compared laminectomy done among 45 patients with the craniotome with footplate technique and 91 patients using the conventional high-speed drill technique [13]. It is possible that with severe lateral recess stenosis from degenerative disease it may be easier for the footplate to catch and tear the dura compared with surgery to treat IMSCT. The indications for the surgeries were not listed; however, based upon the mean patient age of mid-50s in each group, it can be presumed to be largely for degenerative disease. Compared with a literature rate of incidental durotomy for surgery to decompress degenerative stenosis, they found that durotomy occurred in only one (2.2%) patient in the footplate group and six (6.6%) patients in the high-speed drill group, though it was not stated clearly that all the dural tears were caused by the power tool and not by use of any subsequent hand tools.

Based upon the experience described in the case described above, the authors believe that it is important to emphasize that the initial placement of the lip of the footplate must be well-seated under the inferior aspect of the lowest lamina and over the ligamentum flavum. This usually involves creating a small laminotomy with a 3 mm matchstick-type dissecting bit and the 1 or 2 mm Kerrison rongeur on either side that allow for curettes to dissect the attachment of the ligamentum. Mueller et al. noted that the tendency to lift up when progressing upward at the lateral aspect of the lamina must be avoided as this undermines the smooth advancement of the cutting mechanism. In addition, the footplate should not be directed beyond the border of the laminae and facet as this can result in dura and root injury [15]. It is our belief that the complication described in this report occurred when a thoracic root was caught when the footplate was not oriented completely vertical and became directed slightly lateral. When traversing the second trough, a perforating towel clamp or similar instrument may be used to gently provide upward traction to allow the en-bloc laminae to move away from the canal when detached. It is also important to be mindful of the fact that prior surgery and associated epidural scar tissue may impede the smooth movement of the footplate, and thus, the technique is not recommended for revision surgery [15]. It is unclear if trauma, lateral recess stenosis, and chronic epidural steroid injection therapy that may thin the dura might also be relative contraindications.

## Conclusions

Dural laceration is a possible complication of performing a laminectomy using the craniotome with a footplate attachment. To minimize the likelihood of complication, we advise that care be taken not inadvertently allow the cutting mechanism to deviate laterally during the creation of caudal to rostral cuts.
